# 1685. Epidemiological and microbiological characteristics of *S. aureus* pediatric infections in Colombia 2018-2022, a National Multicenter Study: Staphylored Colombia

**DOI:** 10.1093/ofid/ofad500.1518

**Published:** 2023-11-27

**Authors:** Ivan Felipe Gutierrez-Tobar, Cristobal Carvajal, Pablo Vásquez, Jhon Camacho, Joam C Andrade-Fernandez, Juan Pablo Londono-Ruiz, Alejandro Díaz, Cristina Mariño, Miguel Luengas, Derly Hernandez, Jessica F Toro, Angela Niño, Jaime Patiño, Paola Pérez, Lina Sandoval, Rosalba Vivas, Juan Calle, Nancy Cabeza, Eduardo Lopez, Mario Bustos, Yazmin Rodríguez, Paula Araque, María Beltran, Diego Galvis, Juan Lopez, German Camacho, Mayra Jaimes, Claudia Beltran, Natalia Gonzalez, Jinnethe Reyes, Denisse Camacho, Yamile Chaucanes, carlos Garces, Cesar A Arias

**Affiliations:** Clínica Infantil Santa Maria del Lago y Clinica Infantil Colsubsidio, Bogota, Distrito Capital de Bogota, Colombia; Universidad Finis Terrae, SANTIAGO DE CHILE, Region Metropolitana, Chile; STAPHYLORED COLOMBIA, BOGOTA, Distrito Capital de Bogota, Colombia; STAPHYLORED COLOMBIA, HOSPITAL DE SAN JOSE, BOGOTA, Distrito Capital de Bogota, Colombia; STAPHYLORED COLOMBIA, BOGOTA, Distrito Capital de Bogota, Colombia; Clinica Infantil Colsubsidio, Staphylored Colombia, Bogota, Distrito Capital de Bogota, Colombia; HOSPITAL GENERAL DE MEDELLÍN, HOSPITAL PABLO TOBÓN URIBE, MEDELLIN, Antioquia, Colombia; HOSPITAL MILITAR CENTRAL, BOGOTA, Distrito Capital de Bogota, Colombia; HOSPITAL MILITAR CENTRAL, FUNDACIÓN HOSPITAL PEDIÁTRICO LA MISERICORDIA, BOGOTA, Distrito Capital de Bogota, Colombia; HOSPITAL MILITAR CENTRAL, BOGOTA, Distrito Capital de Bogota, Colombia; CLINICA MEDILASER, NEIVA, Huila, Colombia; CLINICA MEDILASER, NEIVA, Huila, Colombia; HOSPITAL UNIVERSITARIO FUNDACIÓN VALLE DE LILI, CALI, Valle del Cauca, Colombia; HOSPITAL UNIVERSITARIO FUNDACIÓN VALLE DE LILI, CALI, Valle del Cauca, Colombia; HOSPITAL UNIVERSITARIO FUNDACIÓN VALLE DE LILI, CALI, Valle del Cauca, Colombia; CLINICA SOMA, MEDELLIN, Antioquia, Colombia; CLINICA FARALLONES, CLINICA VERSALLES, CALI, Valle del Cauca, Colombia; HOSPITAL SAN JUAN DE DIÓS, CALI, Valle del Cauca, Colombia; CENTRO MEDICO IMBANACO, CALI, Valle del Cauca, Colombia; CENTRO MEDICO IMBANACO, CALI, Valle del Cauca, Colombia; CLINICA DEL COUNTRY, CLINICA LA COLINA, BOGOTA, Distrito Capital de Bogota, Colombia; CLINICA DEL COUNTRY, CLINICA LA COLINA, BOGOTA, Distrito Capital de Bogota, Colombia; HOSPITAL SAN JOSE, BOGOTA, Distrito Capital de Bogota, Colombia; FUNDACIÓN HOSPITAL PEDIÁTRICO LA MISERICORDIA, BOGOTA, Distrito Capital de Bogota, Colombia; FUNDACIÓN HOSPITAL PEDIÁTRICO LA MISERICORDIA, BOGOTA, Distrito Capital de Bogota, Colombia; FUNDACIÓN HOSPITAL PEDIÁTRICO LA MISERICORDIA, BOGOTA, Distrito Capital de Bogota, Colombia; STAPHYLORED COLOMBIA, BOGOTA, Distrito Capital de Bogota, Colombia; CLINICA EL ROSARIO, MEDELLIN, Antioquia, Colombia; HOSPITAL INFANTIL DE LA CRUZ ROJA RAFAEL HENAO TORO, MANIZALES, Caldas, Colombia; Molecular Genetics and Antimicrobial Resistance Unit, Universidad El Bosque, Bogota, Distrito Capital de Bogota, Colombia; CLINICA DEL COUNTRY, CLINICA LA COLINA, BOGOTA, Distrito Capital de Bogota, Colombia; Fundación Hospital Infantil Los Ángeles, PASTO, Narino, Colombia; Universidad de Antioquia, Medellin, Antioquia, Colombia; HOUSTON METHODIST RESEARCH INSTITUTE. HOUSTON TX, USA, TEXAS, Texas

## Abstract

**Background:**

*Staphylococcus aureus* infections are a major cause of morbidity and mortality worldwide. Staphylored Colombia is a research network across various regions of Colombia. The present study aimed to describe the epidemiological and microbiological characteristics of *S. aureus* infections in pediatric patients from 2018 to 2021.

**Methods:**

In this retrospective observational study, we analyzed *S. aureus* isolates from centers reported in WHONET, a WHO software for microbiology management. A *S. aureus* pediatric infection (“event”) was defined as any culture isolation in an individual who was previously culture negative for at least 2 weeks. We described center characteristics, age distribution, infection type, and antibiotic susceptibilities. Descriptive statistics were used to compare the microbiologic characteristics of meticillin-sensitive (MSSA) and resistant (MRSA) S. aureus isolates.

**Figure:**
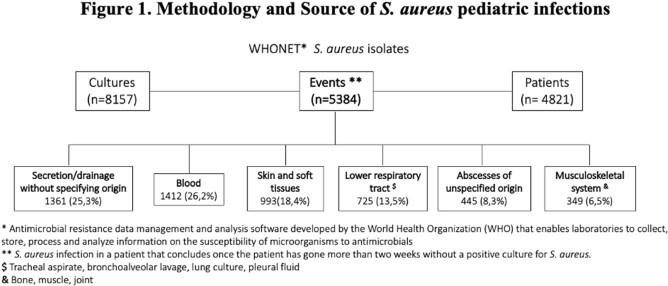

Various origins of S. aureus isolates, according to the approach in methodology: by culture, by event, and by patient

**Results:**

We included 23 centers from 7 cities. Most of these centers (82.6%) provided care for both adults and children, 52.2% offering oncology services and 82.6% having a PICU (Table 1). A total of 8,157 *S. aureus* culture isolations were registered, from 5,384 events that occurred in 4,821 patients (Figure 1). Median age was 5 years (range 1-12). The most frequent infection source was blood (26.2%), followed by skin and soft tissue (18.4%). Most of *S. aureus* isolates remained susceptible to oxacillin (62.1%), clindamycin (85.9%), and TMP-SMX, 91.7%, with minor changes in the antimicrobial resistance overtime (Figure 2). MRSA prevalence varied by city (< 0.001) and was slightly higher in exclusively pediatric hospitals (39.4% vs. 35.7%; p< 0.001), and hospitals with neonatal units (p=0.004). Multiple foci were associated with an increased risk of MRSA (single origin 35.8% vs. three or more origins 56.4%; p< 0.001), particularly in cases of bacteremia (Table 2).

**Figure:**
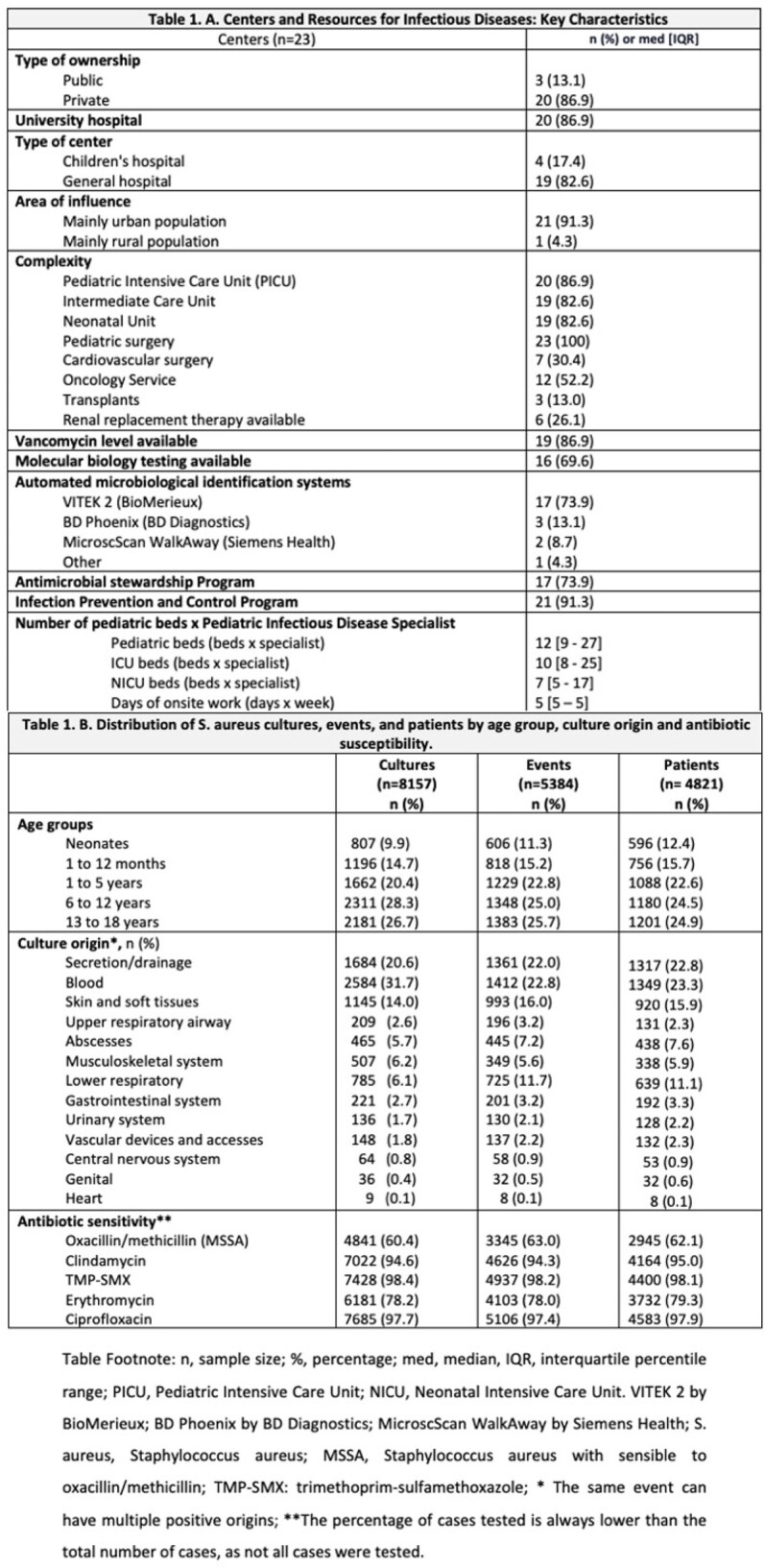

The different characteristics of the centers are described, as well as the description of S. aureus according to the type of isolation: by culture, event, and patient

**Figure:**
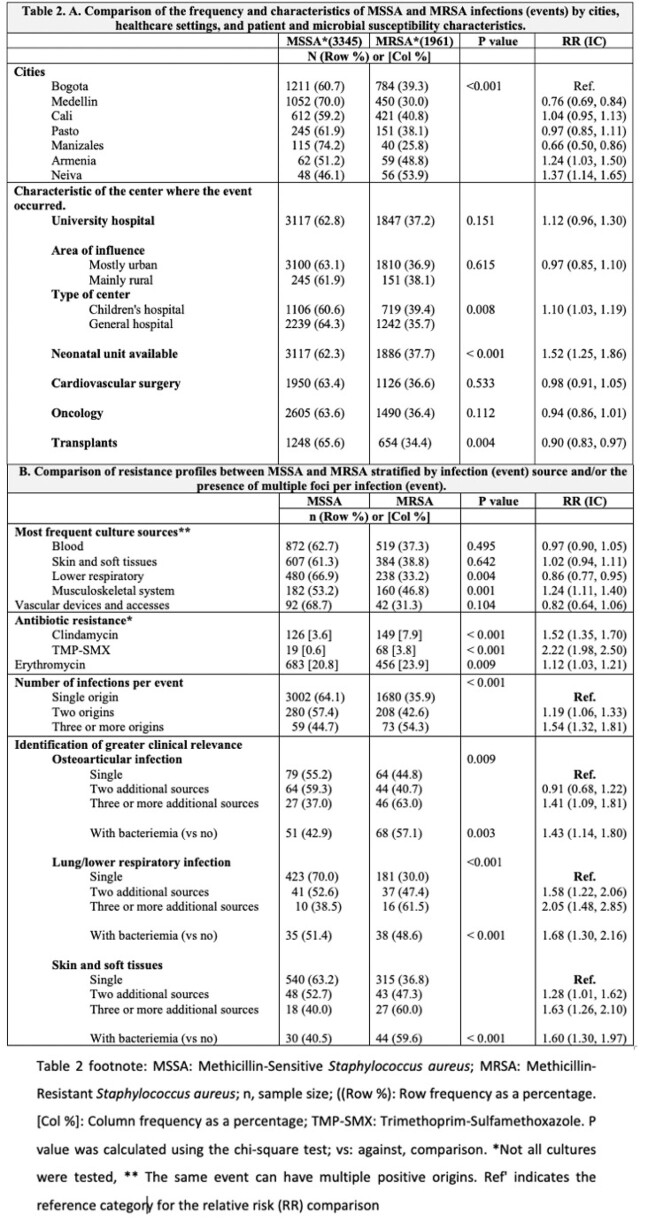

This table compares the SAMS vs SAMR isolates according to the characteristics of the centers and the general type of infection and its origin.

**Figure:**
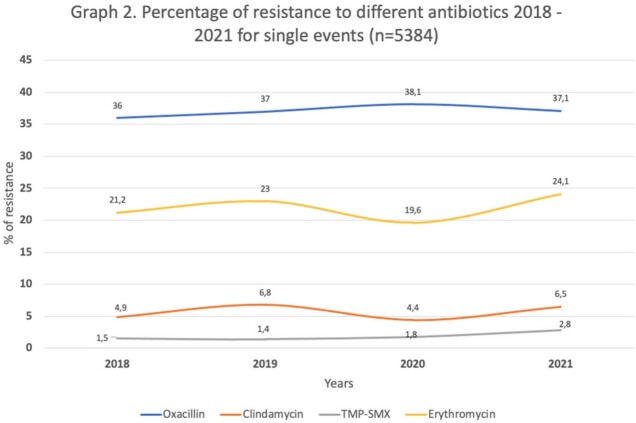

This graph describes how the resistance to different antibiotics for single events has been from 2018 to 2021

**Conclusion:**

Our study, based on WHONET data, suggests that MRSA prevalence varies by region and hospital characteristics. Multifocal involvement and a higher number of infections were associated with an increased frequency of MRSA, particularly in cases of bacteremia. Prospective clinical studies are fundamental and currently ongoing to provide a more comprehensive characterization within the Staphylored LATAM network.

**Disclosures:**

**All Authors**: No reported disclosures

